# Supplementary material for: MetaRibo-Seq measures translation in microbiomes
Source: Nat Commun. 2020 Jun 29;11:3268. doi: 10.1038/s41467-020-17081-z (PMC7324362; doi:10.1038/s41467-020-17081-z)
Supplement: Supplementary file 10 — Supplementary Data 7 [file 41467_2020_17081_MOESM10_ESM.zip › File2/Confidence_High_Taxonomy/32884_out.krona.html]

Javascript must be enabled to view this page.

members
magnitude
magnitudeUnassigned
count
unassigned
taxon
rank

32884\_out

6

superkingdom
2
6

6
phylum
1239

6
class
186801

6
order
186802

5
1898207

SRS013965\_contig\_number\_40001SRS014923\_contig\_number\_38931SRS014979\_contig\_number\_31068SRS053335\_contig\_number\_17119SRS104400\_contig\_number\_31565
species

2039302
genus
1

1
1981510

SRS015217\_contig\_number\_19496
species
